# Antibody repertoire sequencing analysis

**DOI:** 10.3724/abbs.2022062

**Published:** 2022-06-09

**Authors:** Senxin Zhang, Tiange Yang, Xiaojing Liu, Jiyuan Yang, Xiaoqi Zheng

**Affiliations:** 1 Department of Mathematics Shanghai Normal University Shanghai 200234 China; 2 State Key Laboratory of Molecular Biology Shanghai Institute of Biochemistry and Cell Biology Center for Excellence in Molecular Cell Science Chinese Academy of Sciences University of Chinese Academy of Sciences Shanghai 200031 China

**Keywords:** high-throughput sequencing, BCR repertoire analysis, statistical method, profiling and data mining

## Abstract

High-throughput sequencing for B cell receptor (BCR) repertoire provides useful insights for the adaptive immune system. With the continuous development of the BCR-seq technology, many efforts have been made to develop methods for analyzing the ever-increasing BCR repertoire data. In this review, we comprehensively outline different BCR repertoire library preparation protocols and summarize three major steps of BCR-seq data analysis, *i*. *e*., V(D)J sequence annotation, clonal phylogenetic inference, and BCR repertoire profiling and mining. Different from other reviews in this field, we emphasize background intuition and the statistical principle of each method to help biologists better understand it. Finally, we discuss data mining problems for BCR-seq data and with a highlight on recently emerging multiple-sample analysis.

## Introduction

The antigen receptor on B cells, B cell receptor (BCR), recognizes the antigen and plays key roles in B cell development, survival and activation. The secreted form of BCR is also called antibody, which contains two identical immunoglobulin (Ig) heavy (IgH) chains and two identical Ig light (IgL) chains [1]. In the human genome, the *IgH* locus is located at 14q32.33, and *Igκ* and *Igλ* are located at 2p11.2 and 22q11.2, respectively [2]. Both IgH and IgL chains can be divided into the variable N-terminal Ig domain (IgV) and constant C-terminal Ig domain (IgHC or IgLC). The IgV contains highly diversified sequences and is primarily responsible for antigen recognition, while the sequence constant domain can activate downstream immune responses.

In this review, different BCR repertoire library preparation protocols are outlined and three major steps of BCR-seq data analysis, *i*. *e*., V(D)J sequence annotation, clonal phylogenetic inference, and BCR repertoire profiling and mining are summarized.

## Two Layers of BCR Diversification Processes

To recognize different antigens, in each B cell, *Ig* genes undergo two layers of diversification processes to contribute to the total BCR/antibody repertoire, including the antigen-independent and antigen-dependent processes.

V(D)J recombination, occurring during B cell development in the omentum, fetal liver, or adult bone marrow, assembles the *IgV* exon to shape a primary antibody repertoire. In this antigen-independent process, the germline variable (V), diversity (D), and joining (J) gene segments are assembled in an ordered manner from a panel of gene segments spanning in the *Ig* locus. The diversity of primary BCR repertoire comes from the numbers of V/D/J gene segments and is also contributed by the insertion and deletion (indel) at the joining junction [3]. In the IgV domain, the sequence can be further divided into complementarity-determining regions (CDRs) and framework regions (FWRs) [4]. The FWRs and CDR1/2 are contributed by the V gene segment, while CDR3 covers a sequence containing the V-D-J junctions. As a result, the CDR3 is the most diversified sequence and usually plays the utmost important role during antigen recognition [ 5, 6] . From the peptide sequence aspect, CDR3 can be identified from several features, *i*. *e*., starting with a cysteine (C) and ending with phenylalanine (F) or tryptophan (W), as highlighted in the sequence annotation from IMGT (international ImMunoGeneTics information system, https://www.imgt.org/) [7].

Upon antigen stimulation, BCR undergoes another layer of diversification, including IgH class-switch recombination (CSR) and IgV somatic hypermutation (SHM), both of which are initiated by activation-induced cytidine deaminase (AID) [ 8, 9] . In CSR, BCR can switch from IgM to another isotype including IgG, IgA, or IgE [10].While in SHM, AID can initiate mutation or small indels at IgV exons [11]. B cells expressing mutated variable BCRs undergo affinity-based selection in the secondary lymphoid structure named germinal center, leading to a process named affinity maturation [12]. Along with these antigen-dependent processes, naïve mature B cells further differentiate into either antibody-secreting plasma cells or memory B cells [13]. In this context, the B cell pools express an advanced antibody repertoire containing highly potent antibodies of different classes.

## Preparation of BCR Repertoire Library

**Figure 1:**
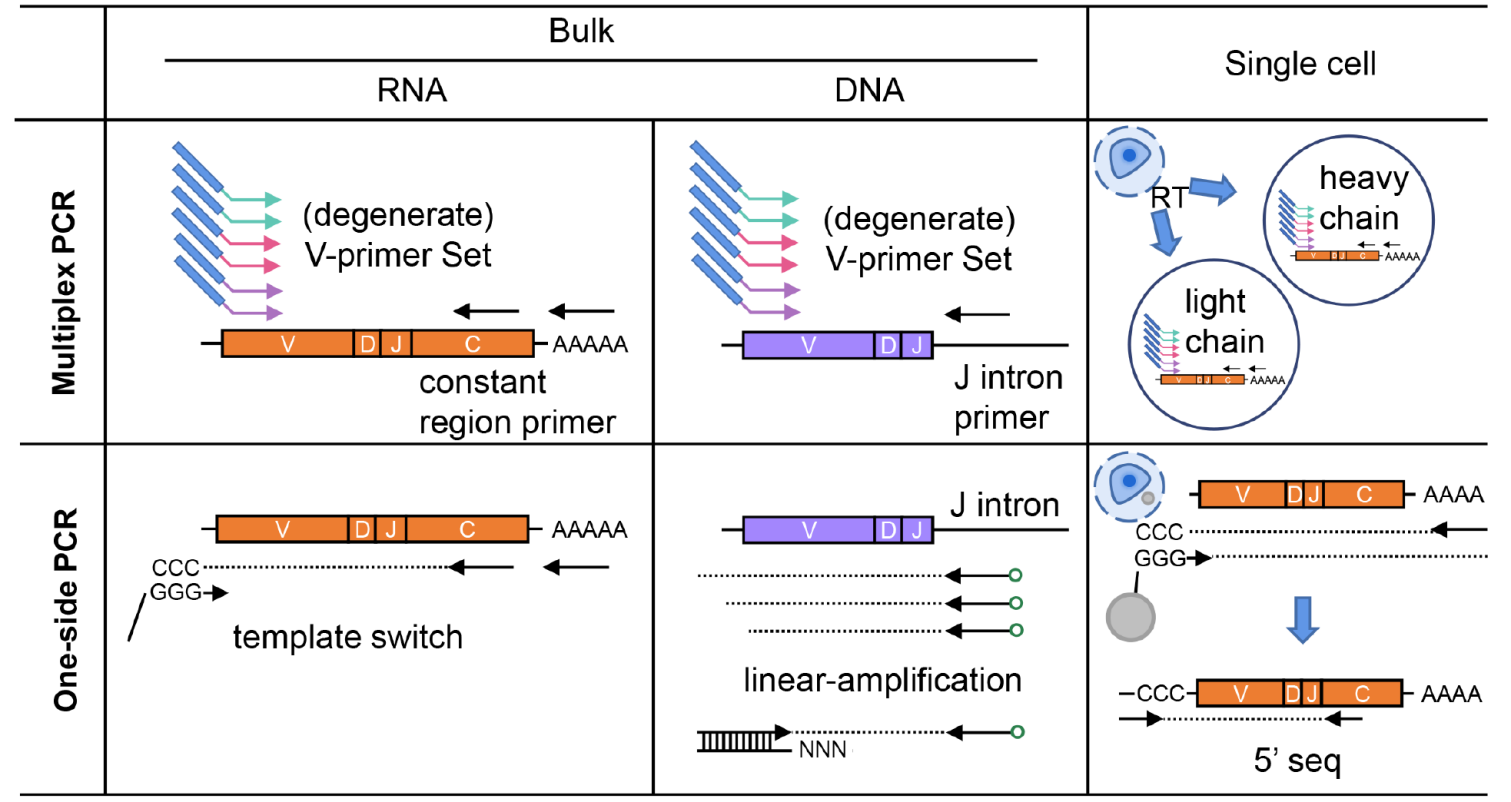
Figure 1 Methods for BCR repertoire preparation Multiplex PCR: regardless of the template types, degenerated primer sets targeting the V gene segments are adopted. Different colors of arrows symbolizes different primer sets targeting various V classes. When mRNA (from bulk or single cell) is used as input, reverse primers annealing to C segments or poly-A sequence are commonly used to amplify the V(D)J. While when genomic DNA is subject to library preparation, the reverse primers usually anneal to the J intron region. One-side PCR methods: In the context of using bulk mRNA as template, after reverse transcription, several cytidines are over-hanged to the 3’ end of synthesized cDNA. Then a “template switch” primer with poly-G sequence will add adaptor on this side. In bulk mRNA samples, 5’ RACE is a popular example. The biotinylated primers annealing to J intron segment are used to select the target ssDNA sequence with streptavidin. In the next step, a dsDNA “bridge primer” is used to mediate the following ligation and synthesize of the other strand. The one-side PCR strategy of single-cell samples, e.g., 5’ seq, is similar to that of bulk mRNA, and the “template switch” primers are attached to a barcoded bead for discrimination between different cell samples.

To present a landscape of BCR repertoire, researchers have developed diverse approaches to prepare such a library ( Figure 1), which can be grouped based on the input materials (bulk or single cell, genomic DNA or mRNA) and cloning strategies (multiplex PCR or one-side PCR).

### RNA from a bulk of cells

The mRNA from a pool of B cells can be subject to library preparation with a multiplex PCR strategy. The complementary DNA (cDNA) can be prepared with a random hexamer or primers annealing to the *Ig* gene constant exon. Degenerated primer sets targeting the V gene segments [ 14, 15] are applied. Along with the development of the method, many efforts have been made to minimize the multiplex PCR bias. On the other way, the one-side PCR method, e.g., 5’ Rapid Amplification of cDNA Ends (5’ RACE), was also applied to amplify the V(D)J sequence in an unbiased way, as exampled in [ 15– 17] . Regardless of the PCR strategy, using RNA as the input complicates the downstream quantification analysis as mRNA levels are not directly correlated with the numbers of B cell clones [18], as BCR mRNA transcripts vary among different B cells. To present a relatively quantitative profile, several strategies can be applied. For example, in one approach, the authors separated the B cells into different aliquots before extracting RNA [ 19, 20] . In another, a unique molecular identifier (UMI) is introduced to tag RNA molecules during cDNA synthesis [21].

### DNA from a bulk of cells

When genomic DNA was used as an input, both multiplex and one-side PCR can be applied. In multiplexed PCR, forward primer sets annealing to the V segments and reverse primers annealing to J segments are usually used [14]. Linear amplification-mediated one-side PCR (LAM-PCR) is used to amplify the V(D)J fragments [ 22, 23] . In this context, DNA-input repertoire allows analysis of both productive and non-productive V(D)J rearranged products, while the mRNA of no-productive allele is degraded through a nonsense-mediated mRNA decay pathway [24] and less-covered in RNA-input library. Furthermore, the BCR sequence number is correlated with the B cell numbers, allowing a more precise evaluation of B cell clonal expansion. However, the DNA-input repertoire losses the IgH class information, as the constant exon is several kilo-base pairs from the V(D)J exon [25].

### Single cell

The emerging single-cell methods enable the analysis of BCR at the single-cell level. Multiplex PCR can be apply to a single B cell to amplify the IgH and IgL chains [26], as exampled by the cloning of anti-viral neutralizing antibodies [27]. On the other hand, 5’ single-cell RNA sequencing (5′ scRNA-seq), as a one-side PCR-based method, utilizes microfluidic devices to profile the antibody V(D)J sequence together with the transcriptome. Compared to the repertoire library of bulk-input, single-cell BCR repertoire retains the IgH and IgL paring information but is low-throughput and costly.

## Analyses of BCR Repertoire Sequencing Data

**Figure 2:**
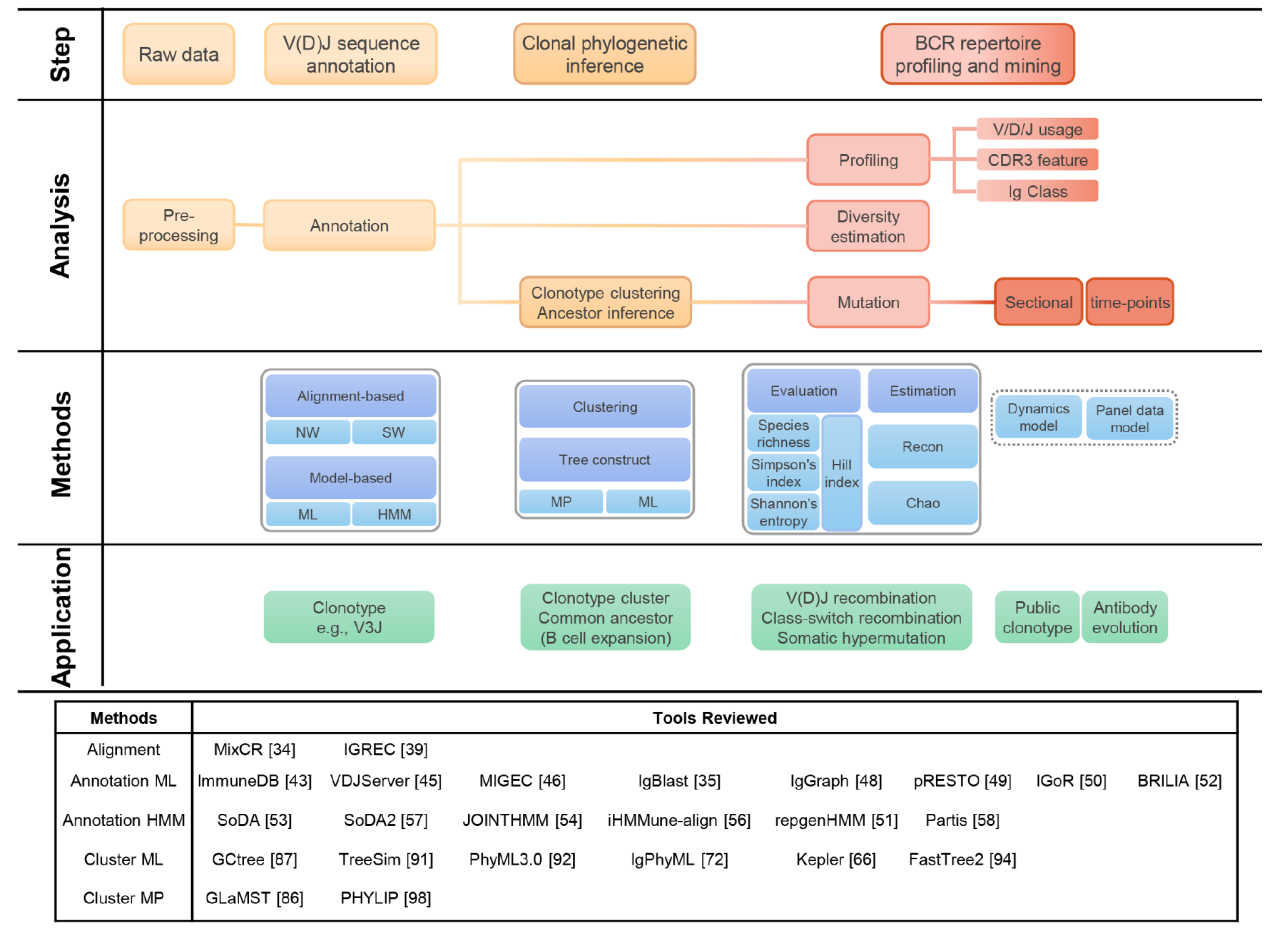
Figure 2 A reference analysis pipeline for BCR-seq data

The BCR repertoire sequencing (BCR-seq) libraries are usually sequenced through high-throughput sequencing (HTS) to generate data in FASTQ format. Different from other types of HTS data, the BCR-seq data analysis can be summarized into the following three major steps [ 28– 30] : (1) V(D)J sequence annotation; (2) clonal phylogenetic inference; and (3) BCR repertoire profiling and mining ( Figure 2). In the following section, we will review the key ideas and their statistical principles in each step.

### V(D)J sequence annotation

As the first step, V(D)J sequence annotation infers the V(D)J gene segments and CDR3 nucleotide/amino acid sequences from the preprocessed BCR-seq data. Generally, there are two ways to annotate the sequence: alignment-based and model-based algorithms, both of which use germline Ig sequences as reference. In this task, IMGT [7] is the most frequently used database for Ig reference.

#### Alignment-based algorithms

Sequence alignment, an algorithm to compare two or more biological sequences, is probably the most fundamental procedure in data analyses [ 31– 33] . It was intensively used in many applications such as functional annotation of an unknown protein/DNA sequence, phylogenetic analysis, and database searching [ 32, 34, 35] . According to different optimization objectives, sequence alignment can be divided into the following two types: global algorithm (Needleman-Wunsch, NW [36]) and local algorithm (Smith-Waterman, SW [37]).

Needleman-Wunsch alignment algorithm aims to calculate the best overall similarity score between the query sequence and a target sequence by a dynamic programming algorithm. On the contrary, the Smith-Waterman algorithm does not devote to comparing the entire sequence, but to finding the fragments with high similarity in two sequences. Both algorithms are successfully applied to DNA and protein sequence analyses, while the local algorithm is more frequently used in BCR-seq analysis. For example, Bolotin et al. [34] proposed MixCR, a comprehensive framework of adaptive immunity analysis with features of low-quality sequence rescue and cluster-based clonotype inference. It uses Subread [38], a local alignment algorithm to annotate the V(D)J gene segment of each core sequence. Another annotation tool in the same category is IGREC [39], which performs a two-step alignment to find the longest subsequence of k-mers between reads and Ig segments to infer the Ig segment, *e.g.*, Ighutil [40]. The annotation step yields the antibody clonotypes. For example, the cAb-Rep database [41] summarized a total of 267.9 million IgH and 72.9 million IgL clonotypes annotated by the SONAR pipeline [42].

#### Model-based algorithms

Due to the complexity of the BCR repertoire, researchers further introduced statistic-based methods to precisely annotate V(D)J sequences. Two types of models are frequently used in this category, *i*. *e*., classical probabilistic model and Hidden Markov Model (HMM).

##### Classical probabilistic model

Classical probabilistic model describes the biological process by a series of dependent or independent probabilistic events, and parameters can be estimated by maximizing the likelihood function of the observed data. This kind of model is widely used in many tools including ImmuneDB, VDJServer, and MIGEC. Among them, ImmuneDB [43] is a tool for adaptive immune repertoire analysis and repertoire data storage. Besides the alignment-based method, ImmuneDB also provides the likelihood of closely related V genes [44]. Two other probabilistic models VDJServer [45] and MIGEC [46] use a model-based searching tool called IgBlast [ 35, 47] to perform the annotation, and IgGraph [48] uses colored de Bruijn graphs to help the annotation. pRESTO [49] is also a model-based annotation scheme that labels individual reads by extending the sequence descriptions.

We take IGoR as an example to illustrate the classical probabilistic model in detail. IGoR [50] models the antibody recombination as three basic types of recombination events, *i*. *e*., germline Ig segments choice, insertion, and deletion (without consideration of mutation events). These interconnected events were described as a conditional probability density function, which is also applied by other tools [51].

For each V(D)J sequence *x*, denote the objective function of it as *P*_rcomb_( *x*, *θ*), where *θ* represents the set of parameters to be determined. When obtaining the value of *θ*, each query sequence can be easily annotated as the V(D)J gene segment that gives the highest observation probability. In detail, it first performs a local or global alignment between the query sequence *x*_*i*_ and the germline gene sequence *g _j_*. Second, it marks each nucleotide as one of the basic types (germline Ig segments choice, insertion, and deletion) of recombination. Third, IGoR substitutes the parameters ( *θ*) and observed values from the last step into the objective function so that we would get the probability *p _i·j_* (the query sequence *x*_*i*_ is expanded from the germline gene sequence *g _j_*). Fourth, IGoR repeats the above steps until all the germline gene sequences are traversed. Then the query sequence *x _i_* will be annotated as the germline gene sequence g _j*_ with the highest probability p _i·j*_ among all *j* germline gene sequences.

The key step of this method is how to optimize the parameter *θ*. Parameter *θ* can be obtained from previous experiments or estimated with the following procedure (for most cases). Based on available observations, one can calculate the likelihood function based on the probabilistic model and maximize the likelihood function to find the best parameters, and the difference between probabilistic and likelihood models is described in Box 1 ( Supplementary Data). IGoR utilized Expectation Maximum (EM) algorithm to calculate the MLE estimation of parameter. The EM algorithm is a powerful statistical method in biological data analysis with latent variables, which is shown as a “coin” example in Box 2 ( Supplementary Data). Briefly, the recombination event (latent variable) is an analogy to the side of “coins”, and the probability of head-side up (the observable variable) is affected by parameter *θ*. After repeating the E and M steps iteratively, both parameter *θ* and recombination events will be obtained. Once we have the parameter *θ*, recombination events of other homogeneous samples can be inferred with ML- or Bayesian-based method directly.

Due to the huge amount of reads in a typical BCR experiment, a classical probabilistic model usually takes substantial computation time. In order to save the computation time and reduce the inference error, other researchers claimed to cluster the sequences first and annotated sequences in the same cluster as one clonal type [52].

##### Hidden Markov Model

Hidden Markov Model (HMM) is another popular annotation algorithm implemented in SoDA [53], JOINTHMM [54] (used in [55]), iHMMune-align [56] and SoDA2 [57]. According to HMM, each reference germline sequence corresponds to a hidden state, which could not be directly observed in BCR-seq data. In contrast, the observed BCR-seq sequences, which undergo recombination events, correspond to the *observed series* (at each position, the observation value correlates with the *hidden state* ). The entire modeling process is named as Markov process. Different from classical probabilistic models, HMM is built up with two probability matrices: *transition matrix* and *emission matrix*.

For one germline sequence, each nucleotide of it corresponds to a hidden stage, which will ‘emit’ an observation among A, T, G, and C. In particular, if the *hidden stage* of a nucleotide is C, it will mostly emit a C, but it will also emit to other nucleotides such as T with a small probability, indicating a C>T mutation event. In the *transition matrix*, each entry quantifies the transition probability of the *i*-th *hidden stage* to the ( *i*+1)-th *hidden stage*, which can be understood as a simple Markov property. In BCR-seq analysis, the indel events further complicate the model from classical HMM. For deletion events, each *hidden stage* can either be transited from the initial state or skip all stages behind it to the next gene segment. Naturally, the closer to the start (or end) of gene segment (J or V) that stage position is, the more probably the deletion event would happen. For insertion events, an N-region topology is inserted at the junction site, which is made up of four stages (A/G/C/T) and forms a self-transition structure. Thus, the *transition matrix* defines the pair-wise transition probabilities between two nucleotides and a loop-break probability at each position.

There are several ways to improve the classical HMM model, e.g., one can use a blank stage between gene segments to block the dependence of cross-segment probabilities (cross V, D, J) and decrease the computing complexity [ 51, 58] ). Alternatively, one can first use the alignment-based method to initialize the parameters of HMM, then apply the model with current parameters to other samples [59].

### Clonal phylogenetic inference

Upon antigen stimulation, B cells undergo clonal expansion in germinal center reaction and are selected by their affinities [60], leading to antibody affinity maturation or antibody evolution (in the long run). Thus, methods for constructing B-cell lineage (phylogenetic tree) and inferring the common ancestor (the root of the tree) are needed. The methods for phylogenetic inference [ 29, 61– 64] mainly solve two issues: (1) clustering, to determine the clonotype scope of a certain phylogenetic tree by a given distance measurement; (2) inferring the ancestral sequence as the root node of the tree, to convert the undirected tree to a directed one. These two issues can be solved separately or integrated [ 64– 66] .

#### Clustering

Clustering is an unsupervised learning method to divide the unlabeled data into several classes and thus can show the unknown data structure and topology. In BCR-seq analysis, clustering is the key step to define a clonotype cluster that potentially represents B cell clonal expansion from one ancestor. As previously demonstrated, the highest abundance antibody usually is not a high-affinity antibody in a BCR repertoire [67]. That’s why we prefer to do clustering for additional valuable information rather than only considering the abundance.

##### Two steps of clustering

Clustering is usually performed in BCR-seq analysis in both annotation and phylogenetic inference steps. In BCR-seq, both PCR-amplification and sequencing steps could introduce errors [ 39, 48] . In order to reduce the systematical error, in the above “V(D)J sequence annotation” step, an extremely strict cluster strategy is frequently applied to infer antibody clonotype. A clonotype is represented by the core sequence to produce more confident results [ 34, 48] , which also helps to reduce the computational complexity as millions or billions of antibody sequences are produced in a BCR-seq experiment [ 45, 68] . Meanwhile, in the clonal phylogenetic inference step, a relatively loose cluster strategy is applied to infer the clonotype cluster. In addition, different from other data, BCR-seq data are characterized by a huge number of categories with small sizes. In graph theory, these data could be described as a large graph with many dense sub-graphs [69], which requires customized clustering methods.

##### Clustering strategy

Clustering algorithms can be roughly divided into the following five groups, *i*. *e*., partition-, model-, density-, hierarchical-, and spectral-based. Basically, all methods aim to find a partition of samples in a way that samples from the same clusters are as similar as possible, while those from different clusters are as different as possible. Many classical algorithms were developed in each group. For example, in single-cell transcriptome data analysis, hundreds of clustering algorithms were developed, most of which require explicit specification of the cluster number *k* [70]. In a typical single-cell transcriptome analysis, the cluster number *k*, which indicates the number of cell types, is usually less than 100 [70]. But in BCR-seq analysis, a fixed *k*is often infeasible for B-cell repertoire clustering due to the huge number of clonal types [69].

Most clustering algorithms require a distance matrix as the input. Distance between two sequences is calculated based on their similarity or alignment score of either nucleotide or amino acid sequences. Nucleotide sequences could contain mutation hotspot information, while amino acid sequences have a much clear biological significance [ 71, 72] . At the nucleotide level, 90% to 95% similarity threshold of CDR3 was mainly used to define a cluster [ 73– 78] . For the whole V(D)J sequence, the 97% similarity threshold is preferred [ 79, 80] . Moreover, other researchers proposed a hybrid threshold of 97% similarity for the whole sequence and 90% similarity for CDR3 [75]. At the amino acid level, most algorithms used identical sequences or with only 1-2 mismatch as a threshold to define clusters in CDR3 [ 19, 79, 81– 83] . An exception was adopted by Meng *et al*. [84] who used a low similarity threshold of 85% in CDR3, *i*. *e*., 2.4 mismatch in a CDR3 of 16 aa.

Hamming distance and Minimum edit distance (MED) are usually used to define the distance between two sequences. Hamming distance is the simplest algorithm that only counts the number of mismatches in the alignment [ 73– 75, 77, 78] . MED is also called the substitution model, integrating mutation, insertion, and deletion events through a quantitative model [ 85, 86] . MED method has been frequently used by GLaMST, TraCeR, and other tools [ 83, 85, 87, 88] . There are also some heuristic distance metrics based on sequence alignment. For example, BRILIA [52] adopted a penalty algorithm to increase the distance accumulation for consecutive mismatches.

##### Strategies to accelerate clustering

The distance matrix requires a great deal of computing resources especially for the high-throughput BCR-seq data. In practice, this problem can be alleviated by either using an approximate algorithm or further dividing the dataset into subgroups. For sub-grouping methods, clonotypes can be grouped based on the combination of V and J gene segments or sequence length. Other methods in this line include IgRepertoireConstructor, which constructs a Hamming Graph with many sub-graphs that are defined by a threshold of hamming distance [69]. A few methods apply approximate algorithms, *e.g.*, IGREC [39] uses a fast minimizers algorithm to find the longest subsequence of *k*- *mers* between reads and germline sequences as a new filtration strategy to cluster sequences.

#### Inference of common ancestor

Maximum likelihood (ML) and maximum parsimony (MP) are the most popular methods to construct a phylogenetic tree inside a clonotype cluster and infer the common ancestor, which was originally developed in evolutionary biology [89]. ML method attempts to construct a tree with the highest probability [64], while the MP method puts its effort into minimizing the number of mutation events (sum of all edge weights).

##### Maximum likelihood method

The ML method is used in both classical probabilistic model-based annotation and phylogenetic tree construction. We take GCtree [86] as an example to show the basic framework of the ML-based method for phylogenetic tree construction. GCtree uses both distance and abundance information to construct a phylogenetic tree. Two key parameters *p* and *q* are introduced, where *p* defines whether the node bifurcates, and *q* defines whether the descendant contains a mutant. These two parameters were assumed as independent in calculating the likelihood of a GCtree, which is defined by multiplying the likelihood function of each node (clonotype) [86]. At last, the infinite type of assumption is raised to make sure each node can be identified with one sub-tree in the original lineage tree. An EM algorithm is applied to estimate the parameters, while the tree topology is treated as a *latent variable* , and the parameter θ =( *p*, *q*) as **observable variable*s *. Using the initial tree topology and the observed abundance, we can get the estimated parameter $\overset{⌢}{\theta }$ . Based on $\overset{⌢}{\theta }$ , the user can reconstruct the tree and perform another iteration of estimation.

Tools for inferring phylogenetic tree by the ML framework based on different model assumptions. For example, TreeSim [90] generates a lineage tree by modeling the extant species evolution process as a dynamic method––episodic birth-death process (EBDP). PhyML3.0 [91] utilizes the nearest neighbor interchanges (NNIs) to get a fast approximate MLE. Bonsignori *et al*. [92] used PhyML3.0 to construct a phylogenetic tree and infer the common ancestor of the VRC01 anti-HIV-1 antibody lineage. Paschold *et al*. [87] used a fast approximate MLE method, FastTree2 [93], to infer the phylogenetic tree of SARS-CoV-2-specific antibody. Though not as accurate as ML-based methods, it is 100-1000 times faster. IgPhyML [71] estimates a transition rate matrix by considering the hot-spot and cold-spot motifs and other situations. Kepler’s group developed a computational framework that iteratively infers a phylogenetic tree by taking the unmutated ancestral sequence with the highest posterior probability and re-annotate the sequence set [66]. It can integrate the VDJ recombination and phylogenetics through the iterative algorithm.

##### Maximum parsimony method

Maximum parsimony (MP), also called the minimum spanning method, is an approximate algorithm for phylogenetic tree construction. The main procedure of MP usually starts with inferring an undirected tree by a certain distance metric and is followed by an iterative step of trimming and rewiring the phylogenetic tree. The undirected tree can be constructed by the Kruskal’s algorithm [94] or Prim’s algorithm [ 95, 96] , which are based on edges and nodes, respectively. They sort the edges by their weights (or nodes’ distance in Prim) and continuously choose the edge (node) that keeps the graph acyclic in order to construct a minimum spanning tree (MST).

Some existing MP-based tools, such as GLaMST [85], use a heuristic method to literally trim and rewire the lineage tree and complete the tree by generating the intermediate sequences that were not observed. Liberman *et al*. [97] used PHYLIP, an MP-based method to produce a lineage tree and detect population selection [98]. Li *et al*. [76] constructed an undirected tree and used it to analyze lymph node IgA-expressing.

These two types of methods have their advantage and disadvantage. ML methods can bring the best result under its model assumption, while MP methods have a significant advantage in computation time. For both types of methods, we can use the conservative tree estimation process to obtain a more confident result of both the structure of the tree and other analyses such as substitution model. For instance, we can exclude nodes without descendants or just remove trees with only a two-tier structure.

### BCR repertoire profiling and mining

The BCR-seq data contain multiple layers of information, which can be displayed in various ways. Here, we review the popular single-sample profiling approaches and also introduce the emerging profiling and data-mining approaches for multiple-sample analysis.

#### Single-sample analysis

Outcomes of BCR-seq analysis mainly include the following categories: profiling, diversity estimation, mutation frequencies (substitution model), similarity analysis to known antibodies, public (shared) clonotypes, convergent among samples, and so on [ 29, 64, 99, 100] .

##### Data display

BCR repertoire can be profiled in the following ways. First, the V, D, and J gene segment usage (distribution) can be visualized by using a simple bar plot or scatter plot as well as the Ig class [ 101, 102] . The sequence features, like nucleotide or amino acid motifs, can be shown with a logo plot [103]. Meanwhile, the distribution of CDR3 length, clonotype abundance, or amino acid charge can be displayed [ 68, 77, 104– 107] . In a clustered tree, the substitution model can be applied to estimate the frequency of mutation and indel generated in V(D)J recombination and somatic hypermutation (SHM) processes, which is an important problem in BCR repertoire analysis [ 71, 72] .

##### Diversity evaluation and estimation

Diversity of a BCR repertoire is an important feature of antibody diversification. Traditionally, diversity can be evaluated with three commonly used indexes: species richness, Simpson′s index, and Shannon′s entropy [108], which provide different aspects of abundance partition. For example, species richness is the total number of species, which can be simply an analogy to clonotype richness. Simpson′s index reflects the abundance of dominant species in a certain sample, and is regarded as an index named “dominance concentration” [109]. The Shannon index quantifies the uncertainty in the species identity of an individual that is randomly picked from the dataset [110]. Those indexes reflect incomplete information of diversity. Hill [108] integrated them and gave a unified index–Hill index, which covers all of the above three indexes as special cases. The Hill index is suggested as the “true diversity” index by many researchers [ 110– 115] . To compare the diversity among multiple samples ( *evaluation problem*), the parameter *q* in the Hill index can be set as 2 or +∞. To infer the diversity of the whole repertoire ( *estimate problem*), *i*. *e*., the total number of clonotypes, the parameter *q* in Hill index should be set as 0. For example, Bashford-Rogers *et al*. [101] used the Gini index to evaluate the unevenness of the number of RNA molecules, while Galson *et al*. [116] evaluated the repertoire diversity by Shannon index. For estimation problem, published studies have explored the relationship between sequencing depth and observed clonotype number, and used Recon [117] or Chao [ 118– 120] estimator to estimate the missing number of unique clonotypes [ 14, 68, 76, 121] .

#### Multi-sample analysis

Currently, the BCR-seq technology is widely used in profiling the different BCR repertoires from multiple individuals or samples collected at different time points. However, tumor-infiltrate BCR repertoires are found to be heterogeneous and convergent among different cancer types, healthy degrees, and pathological stages [ 87, 122] . Discovering the shared clonotypes among different individuals is a useful way to further analyze BCR repertoire [ 68, 123] . For example, after comparing the numbers of common clonotypes in different samples with the random simulation samples, Soto *et al*. [121] found that the overlapping clonotypes in the human BCR repertoire are at a higher level than the simulation one. Computing the public clonotypes among samples from different individuals [68] is a useful way to further analyze BCR repertoire.

In clinical scenarios, multiple samples can be either time-point samples or sectional samples. The former corresponds to samples of an individual in different time points, presenting a dynamic profile. The latter refers to samples from different individuals at one time point. For example, time-point samples can be obtained in vaccine research, and the BCR dynamic profiles can be applied to predict vaccine response. For sectional samples, public clonotypes could reveal a convergent response, which was reported in several studies [ 61, 124, 125] .

## Perspective

In the past few decades, many efforts have been made to the annotation and phylogenetic inference for BCR repertoire analysis. With the continuous development of the BCR-seq technology, methods for extra-large data set are urgently needed. Compared to other types of biological big-data, BCR-seq data need more stable and accurate statistical models to describe the BCR repertoire in a quantitative way. Due to the large sample size (number of BCRs) in a typical experiment, deep learning models are promising to participant in different tasks of BCR-seq data analyses and mining. For example, Chen *et al*. [126] used T cell receptor diversity data to train a multimodal recurrent neural network to predict the likelihood of antigen presentation. Tang *et al*. [127] developed DeepSHM, a deep convolution neural network (CNN) model to identify extended motifs of SHM beyond hotspot targeting. Since deep learning can solve the problems of classification, clustering, regression, and pattern recognition in other fields, making it is suitable to tackle the problems in immune profiling. We could anticipate more comprehensive applications of deep learning in BCR-seq data analysis in the near future.
